# Prediction of *Escherichia coli* expression performance in microtiter plates by analyzing only the temporal development of scattered light during culture

**DOI:** 10.1186/s13036-017-0064-5

**Published:** 2017-07-03

**Authors:** Tobias Ladner, Martina Mühlmann, Andreas Schulte, Georg Wandrey, Jochen Büchs

**Affiliations:** AVT - Aachener Verfahrenstechnik, Biochemical Engineering, RWTH Aachen University, Forckenbeckstraße 51, 52074 Aachen, Germany

**Keywords:** BioLector, *Escherichia coli*, Microtiter plate, Induction optimization, Scattered light

## Abstract

**Background:**

*Escherichia coli* is often used for recombinant protein production. The expression of recombinant proteins negatively affects the microbial growth, thus, a balance between protein expression and biomass formation is preferable to reach high product- and space-time-yield. Already in screening experiments, suboptimal conditions causing too weak or too strong induction must be avoided. High-throughput screening devices such as the BioLector are often applied for screening experiments. The BioLector allows optical online monitoring of each well in a continuously orbitally shaken microtiter plate via scattered light and fluorescence measurements. This technique enables a fast identification of promising clones. However, to determine the expression performance of non-fluorescent products elaborated offline analysis is often required.

**Methods:**

A mathematical method is developed to distinguish between cultures, which are insufficiently, optimally or too strongly induced. Therefore, just the temporal development of the scattered light intensity signal is investigated. It is found that discrimination between the different intensities of induction is possible via principal component analysis. By fitting an extended sigmoidal function to the trajectory of the scattered light over time, two characteristic parameters are found. These are used in an empirical model to predict the expression performance.

**Results:**

The method was established for a wide range of culture conditions based on 625 *E. coli* cultures. Three *E. coli* host strains (Tuner(DE3), BL21(DE3), and BL21-Gold(DE3)) expressing either flavin-mononucleotide-based fluorescent protein (FbFP) or Cellulase celA2 were investigated. Cultures were conducted in two different types of microtiter plates (48- and 96-wells), in two online measurement devices at four temperatures (28 °C, 30 °C, 34 °C, and 37 °C). More than 95% of the predicted values are in agreement with the offline measured expression performances with a satisfying accuracy of ±30%.

**Conclusions:**

The properties of cultures studied can be represented by only two characteristic parameters (slope at and time of the inflection point) received from fitting an extended sigmoidal function to the respective scattered light trajectory. Based on these two characteristic parameters, predictions of the standardized expression performance are possible and for a first screen elaborated offline analysis can be avoided. To the best of our knowledge, this is the first work presenting a method for the general prediction of expression performance of *E. coli* based solely on the temporal development of scattered light signals.

**Electronic supplementary material:**

The online version of this article (doi:10.1186/s13036-017-0064-5) contains supplementary material, which is available to authorized users.

## Background

As early as 1978, genetically modified *Escherichia coli* was used for the synthetic production of human insulin [1]. In the recent past, *E. coli* became one of the most often used prokaryotic expression systems for production of recombinant proteins. This can be attributed to the today sequenced genome [2]. *E. coli* allows for an easy introduction of foreign genes [2–4]. Furthermore, this bacterium can grow fast on low-cost media reaching high cell densities which is advantageous for economical protein production [3].

In the literature, it is known that the production of recombinant proteins can negatively affect the microbial growth by a process often termed metabolic burden [5]. To ensure sufficient biomass concentration for protein production, the culture is often divided into a growth phase and a subsequent production phase by applying controllable expression systems. Such expression systems are externally controlled, for example by temperature shift [6], certain levels of the dissolved oxygen tension [7] or chemical inducers [4]. The most popular inducer molecule in laboratory scale is probably isopropyl β-D-1-thiogalactopyranoside (IPTG). It was shown that the time-point of induction as well as the amount of added inducing compound have significant impact on the culture [8–10]. Thus, it is crucial to find optimal induction parameters revealing a balanced process. Insufficient or too strong induction have to be avoided to achieve high product yield. Unfortunately, optimal induction parameters found for a specific system are not universally valid and, thus, a direct transfer from bioprocess to bioprocess is not possible. The optimal combination of induction time and inducer concentration depend besides other factors on the *E. coli* host strain, the expression plasmid or the recombinant gene [11–13]. For each bioprocess, optimization of the induction parameters has to be carried out. This results in numerous cultivations that have to be conducted. Therefore, small-scale high-throughput screening devices are often applied that allow for cost-efficient studies of parallel cultures [14].

The BioLector is a meanwhile widespread high-throughput screening device which is based on continuously orbitally shaken microtiter plates (MTP) [15, 16]. This technology allows for optical online monitoring of the scattered light and fluorescence in each well of a MTP. If fluorescent proteins like the green fluorescent protein (GFP) and its derivatives or the flavin-mononucleotide-based fluorescent protein (FbFP) are used as fluorescence tag, the product formation is directly accessible by means of online fluorescence measurements. By combining a BioLector device and liquid handling systems it becomes possible to set up a fully automated screening platform (RoboLector) [10, 17]. For example, Huber et al. [10] developed an induction profiling method realizing an automated individual induction depending on the online monitored biomass concentration. By using the “biomass-specific induction”, only small relative standard deviations from well to well were obtained for the expressed FbFP.

However, the use of fluorescent protein tags is usually unwanted in production scale. It is a question whether the result of screen with a fluorescent tag fused to the target protein can be directly transferred to a culture expressing the target protein without fluorescence tag. Rahmen et al. [18] found that already a single amino acid exchange in the recombinant protein and even only a silent codon exchange [13] has an significant effect on the metabolic burden of the *E. coli* host strain. It is therefore, very unlikely that the removal of a fluorescence tag after screening does not affect microbial growth. Thus, changes of the parameters found for optimal induction have to be expected. For quantification of products which are not accessible via fluorescence measurement, samples taken during (or at least at the end of) the culture have to be offline analyzed. Mühlmann et al. [17] presented an extended RoboLector system including an integrated downstream processing unit. Samples for investigation of intracellular and secreted enzymes can be prepared and analyzed with the demonstrated system [17]. For cell separation, a microfiltration unit is used. The activity measurement of the expressed enzymes via assay was not fully included into the automated procedure. Similar systems were used by Rohe et al. [19] and Unthan et al. [20]. They applied centrifugation for cell separation and, furthermore, integrated a photometer to realize fully automated MTP assays [19, 20].

The integration and automation of up- and downstream as well as analytic units require technically sophisticated systems. With each additional device, the overall system becomes more complex, error-prone and requires longer development time. Technical efforts might be reduced by applying more advanced mathematical methods. Recently, a system for online multi-wavelength (2D) fluorescence spectroscopy in each well of a MTP was presented [21]. By applying chemometrics based on the acquired multi-wavelength (2D) fluorescence spectra, models were developed to predict the concentration also of non-fluorescent compounds such as carbon sources and overflow metabolites (glucose, acetate and glycerin) during the cultures [21]. In stirred tank reactors, chemometrics are already applied since longer time. For example, chemometrics are used to monitor spore germination, metabolic activity or monoclonal antibody production [22–24]. This indicates the opportunities of this method. However, chemometrics require deepened mathematical understanding and, therefore, are sometimes difficult to apply by standard users.

In the present work, 625 cultures of three different *E. coli* host strains conducted in two different types of MTPs (48- and 96-well), at four temperatures (28 °C, 30 °C, 34 °C and 37 °C) are investigated with two online measurement devices. An easy to use mathematical method is developed to identify insufficient and too strong induction. Just an extended sigmoidal function has to be fitted to the temporal development of the scattered light signal. Based on the fitting, two characteristic parameters representing the entire culture are derived. Using these two characteristic parameters, an empirical model is developed to estimate a relative expression performance of the corresponding culture. It becomes possible to predict the expression performance of *E. coli* cultures and identify potential improvements without elaborated offline analysis.

## Results and discussion

### Visualizing trends in the temporal development of the scattered light to identify optimal induction

During cultivation, for example the scattered light intensity signal is used to monitor the biomass concentration. Scattered light intensity and biomass concentration usually correlate directly. With increasing biomass concentration, also the scattered light intensity signal is increasing. Concentrations can only be determined by calibration, because the scattered light intensity signal is a semi-qualitative measurement (arbitrary units) [16].

Further information about the investigated process can be obtained based on the temporal development of the scattered light. For example, morphological changes of the cells and the development of sub-populations can be identified by means of changes in the scattered light signal [25, 26]. In the following, patterns of the temporal development of the scattered light acquired during induced *E. coli* cultures compared to non-induced culture are considered to identify a general trend for prediction of the expression performance.

**Fig. 1:**
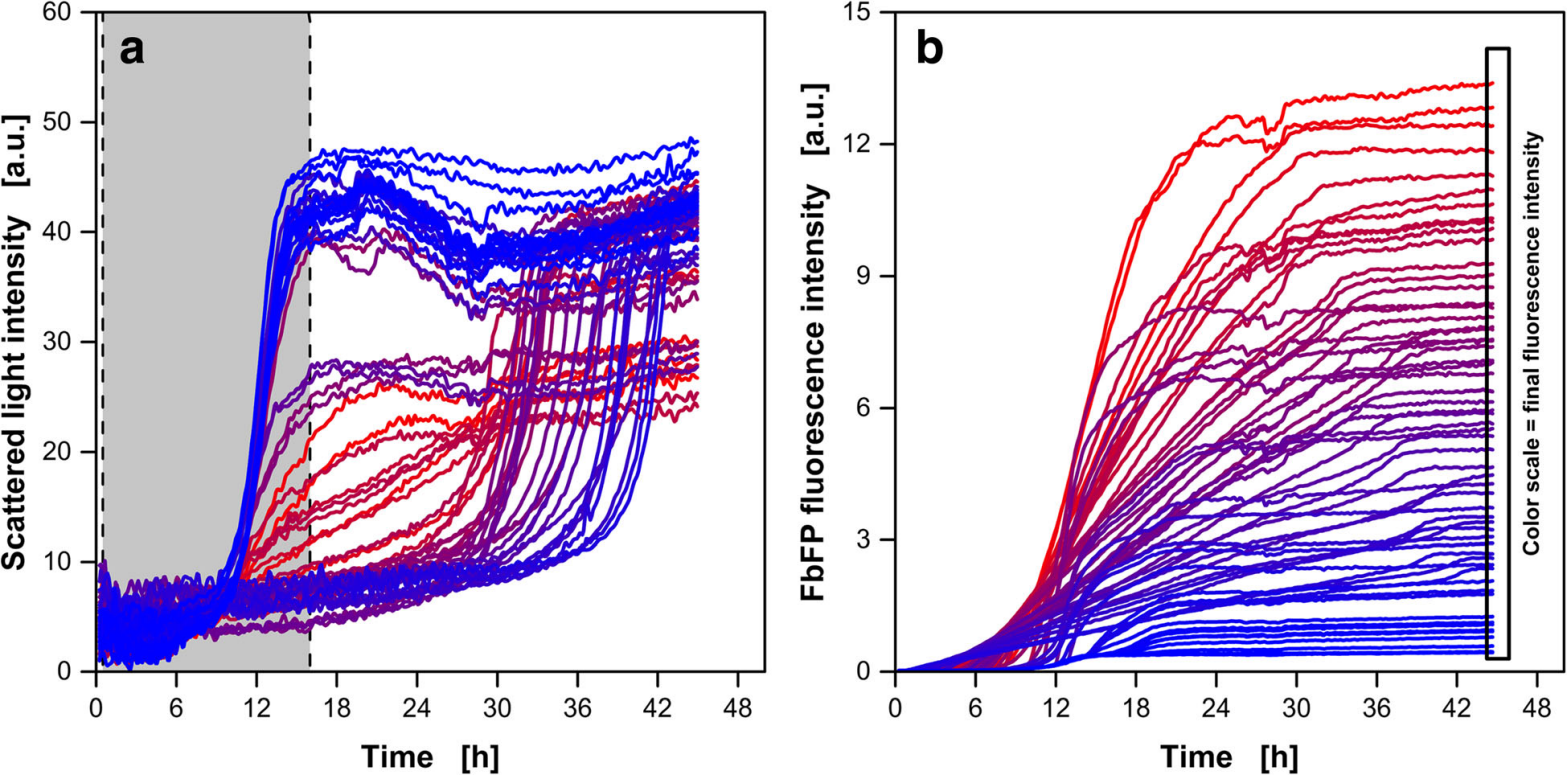
Monitored scattered light (**a**) and FbFP fluorescence intensity signals (**b**) during *E. coli* Tuner(DE3)/pRhotHi-2-LacI-EcFbFP cultures with varying times of induction (0.5–16 h) and concentrations of IPTG (0–1000 μM). **a** The time span of induction is highlighted by the gray area. For clarity, only 61 cultures of the entire data set (303 cultures, Additional file 1: Figure S1) are shown. **b** The final FbFP fluorescence intensity defines the color-coding. The weakest expression (≙ minimum final FbFP fluorescence intensity) is presented in blue and becomes more reddish with increasing final fluorescence intensity. The maximum final fluorescence intensity is presented in pure red. The 303 cultures were conducted in a total of eight MTPs. The investigated cultures are referred to as dataset A in Table 1. Cultivation conditions: 48 round deep-well MTP without optodes, V_L_ = 800 μL, *n* = 1000 rpm, shaking diameter d_0_ = 3 mm, 30 °C

In Fig. 1, cultures of an *E. coli* strain expressing flavin-mononucleotide-based fluorescent protein (FbFP) after IPTG-induction are presented. For clarity, only 61 cultures of the entire dataset (303 cultures) are shown. The online monitored signals of all 303 cultures are given as Additional file 1: Figure S1. The time of induction was varied between 0.5 and 16 h (gray shaded area in Fig. 1a) with a maximum applied IPTG concentration of 1000 μM. Due to the online fluorescence measurement, the FbFP formation is directly accessible for online monitoring (Fig. 1b). A color-coding, based on minimum and maximum final expression performance of the entire dataset (303 cultures), is used in Fig. 1 and Additional file 1: Figure S1. The weakest expression performance (≙ minimum final FbFP fluorescence intensity) is presented in blue and becomes more reddish with increasing final fluorescence intensity. The maximum final fluorescence intensity is decoded in pure red. This color-coding is applied to both, the scattered light intensity signal (Fig. 1a) and the FbFP fluorescence intensity (Fig. 1b). Signals of the same culture are presented with the same color.

It is clearly visible that cultures with more or less linear scattered light intensity increase between 6 and 30 h are the most reddish (Fig. 1a). Accordingly, the highest final FbFP fluorescence intensities is measured at the end of these cultures (Fig. 1b). In contrast, cultures with an exponential increase after 12 h (in the following referred as “insufficient induction”) or cultures with an extended lag-phase followed by an exponential increase of the scattered light signal between 30 and 42 h (in the following referred as “too strong induction”) are mostly presented in blue (Fig. 1a). Cultures belonging to the group “insufficient induction” do not show any significant impact on biomass formation after IPTG addition. Thus, the amount of IPTG added was too low to induce all cells and (almost) unimpeded growth occurred. The extended lag-phase of the cultures belonging to “too strong induction” indicates that the amount of IPTG added was too high or IPTG was added too early to the culture broth. In this case, the metabolic activity was more or less completely shifted to protein expression instead of biomass formation. A low number of cells expressing FbFP are present and, thus, the overall culture time increases significantly. Differences in the temporal development of the scattered light of the three groups also become clear in Fig. 3b-d. In each figure, the scattered light signal of a (non-induced) reference culture (gray dashed line) is shown to allow for direct comparison. The scattered light of a culture belonging to the group of insufficient induction is presented in Fig. 3b (red line). The signal for an optimal induction is shown in Fig. 3c (red line) and too strong induction is represented by the pattern in Fig. 3d (red line).

As mentioned above, the best expression performance was found for the cultures showing a linear increase of the scattered light intensity signal after induction. A linear increase of the scattered light intensity signals represents the best compromise between insufficient induction and too strong induction. This leads to the presumption that optimal induction of *E. coli* cultures is indicated by a (mostly) linear increasing scattered light signal after induction. In the following sections, classification and predictions of the expressions performance by means of the scattered light patterns are investigated on a more statistical and mathematical basis.

### Clustering of *E. coli* cultures via principal component analysis (PCA)

In the previous section, *E. coli* cultures were classified based on the scattered light intensity patterns and their final FbFP fluorescence intensity by applying a color-coding for visualization. Three groups were identified: “insufficient induction” (including non-induced cultures), “optimal induction” and “too strong induction”. To realize clustering following a mathematical approach, principal component analysis (PCA) is often applied [27, 28]. PCA is a statistical method, which converts a set of (probably) correlated variables to new uncorrelated variables (principal component, PC). The first PC represents the largest degree of variance and, thus, carries the greatest amount of information about the dataset. Each following PC is orientated orthogonal to the previous PCs and carries less information than the previous PC. As result, large datasets can often be reduced to score values of just a few PCs. These PC scores are key parameters used for classification.

**Fig. 2:**
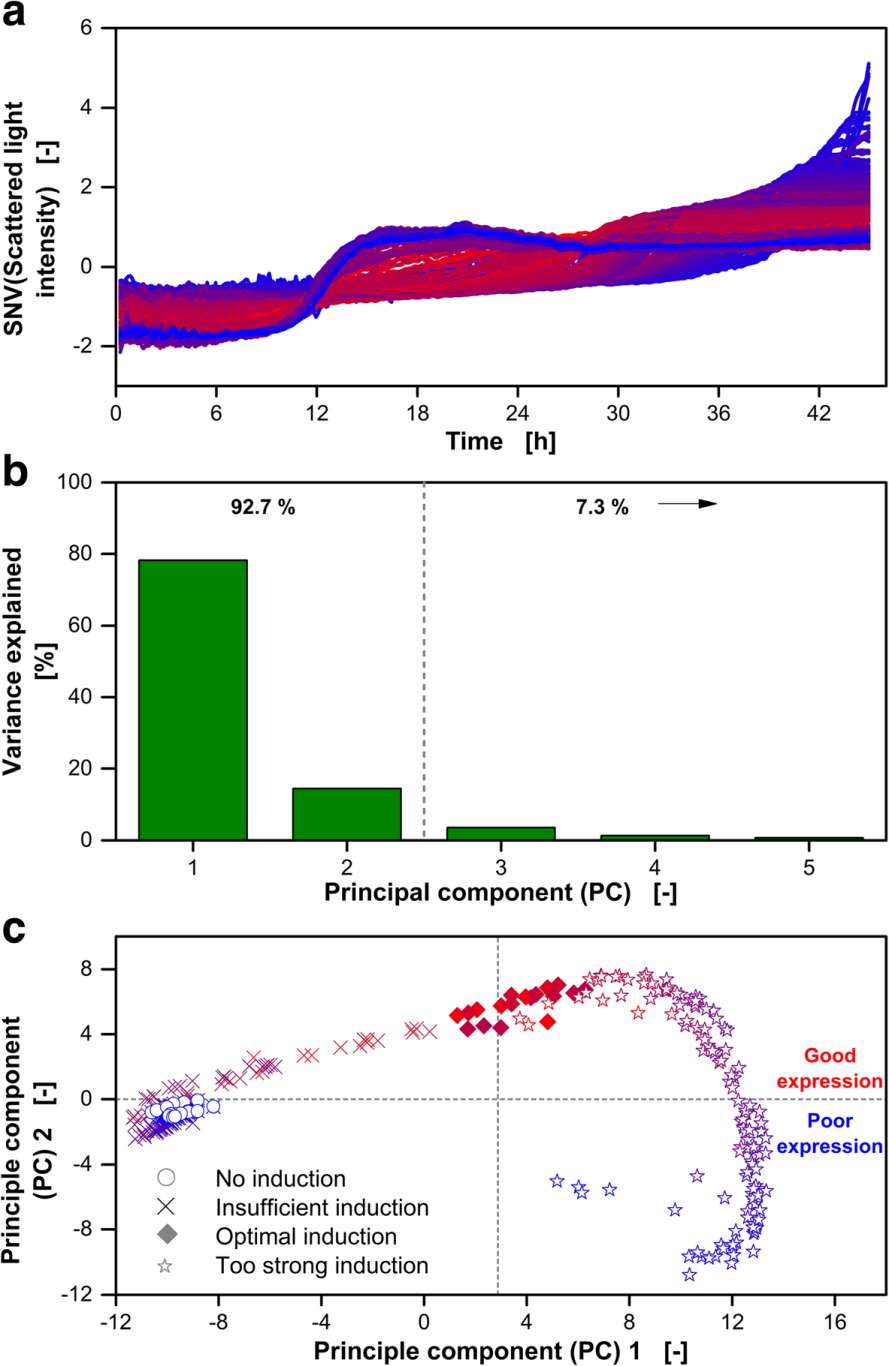
Analysis of scattered light intensity patterns applying principle component analysis (PCA). **a** For data pre-processing, the standard normal variation (SNV) at each scattered light intensity curve was calculated according to Eq. 5. The SNV transformed data of the 303 *E. coli* Tuner(DE3)/pRhotHi-2-LacI-EcFbFP cultures introduced in Fig. 1 was used for PCA. **b** Variance explained by each principle component (PC) of the entire dataset. **c** Scores of the second PC over the scores of the first PC. The color represents the final FbFP fluorescence intensity and corresponds to the color-coding in Fig. 1. The symbols represent the classification of the scattered light intensity pattern: no induction, insufficient induction (pattern close to non-induced culture), optimal induction (more or less linear course in Fig. 2a) and too strong induction (significantly lengthened lag-phase). The classification was performed manually. The investigated cultures are referred to as dataset A in Table 1

Before calculating the PCs, it is recommended to perform scaling and centering to increase the quality of the PCA [29]. Therefore, the scattered light intensity signals are standard normal variate (SNV)-transformed as described in Methods (Eq. 5). The SNV-transformed scattered light intensity values over time of all 303 cultures are presented in Fig. 2a. The color-coding introduced in Fig. 1 is applied. Again, cultures showing linear increase of the scattered light between 12 and 30 h turn out to be the most reddish and, thus, have the best expression performance.

Based on the SNV-transformed temporal development of the scattered light (Fig. 2a), the PCA is calculated. The online monitored FbFP fluorescence intensities are not used for PCA calculation. The first and second PCs account already for 92.7% of the total variance of the dataset (Fig. 2b). The remaining PCs explain only 7.3% of the total variance (Fig. 2b). Consequently, the first two PCs are expected to be sufficient for discrimination between the three above-mentioned groups (insufficient, optimal and too strong induction).

In Fig. 2c, results of the PCA are presented by plotting the scores of the second PC over the scores of the first PC. Each data point represent one entire culture. To make the expression performance of the corresponding culture visible, the color-coding introduced in Fig. 1 is used again (Fig. 2c). Furthermore, classification into insufficient, optimal and too strong induction is indicated by different symbols. Non-induced cultures, which are more or less just a special case of insufficient induced cultures, are highlighted by open circles in Fig. 2c. It becomes clear, that cultures belonging to different groups of induction are locally separated (Fig. 2c). The scores of the first PC indicate the shape of the scattered light pattern. Score values of the first PC smaller than 2 tend to follow unimpeded biomass formation (insufficient induction). Too strong induction is indicated by score values greater than 4 in the first PC. For cultures with optimal induction, score values of the first PC close to 3 are obtained. In Fig. 2c, the score value of 3 in the first PC is highlighted by a gray dashed vertical line. The information contained in the score values of second PC correlates with the maximum final FbFP fluorescence intensity. Cultures having a positive value in the second PC tend to show good expression performance (mostly reddish). In contrast, negative score values indicate a poor expression performance (mostly bluish).

The investigations presented in Fig. 2 clearly show that a classification of *E. coli* cultures based on the temporal development of the scattered light is possible by means of PCA. Only the first two PCs are required. It is very likely that a correlation between the scores of the first two PCs and the expression performance of the corresponding culture can be found. However, there are some drawbacks in using PCA-regression in the present case. The PCA is calculated based on the temporal development of the scattered light, which has discrete time intervals. For the chosen measurement conditions, the temporal development of the scattered light during culture is represented by 448 single scattered light values over time (measurement cycle time of 6 min). If less wells are monitored in parallel, the cycle time can be reduced to increase the data density and more data pre-processing becomes necessary to equalize the time vector. If the culture temperature is reduced, the overall culture time will increase, leading to an extended absolute culture time. This change must also be staved by the data pre-processing. Finally, that would result in a more and more complex procedure to achieve general validity allowing transfer of the PCA-model to other systems. Since standard users are often interested in as simple as possible methods, the mathematical evaluation should not include many pre-processing steps. Therefore, a more mechanistic approach has to be followed which is described in the following.

### Clustering of cultures based on characteristic parameters received from fitting an extended sigmoidal function to the temporal development of the scattered light

In the previous section, just the scores of the first two PCs were required to classify cultures according to the intensity of induction based on their scattered light trajectory. To apply the PCA-model to different systems, identical conditions (culture time, measurement setup and discrete time steps) are essential. Otherwise, a more complex data pre-processing is required. To overcome this restriction a more general procedure, which can be applied to any scattered light (respectively biomass concentration) monitoring device and various measurement conditions, is to be developed.

The application of a (purely) mechanistic model based on Monod kinetics is not possible, because information about substrate and (potential) overflow metabolite concentrations during culture is not accessible and required kinetic parameters such as the biomass yield coefficients are unknown. Furthermore, the effects due to induction are difficult to represent by Monod kinetics and the resulting model as well as the evaluation method would become more complicated. Instead, a simple four-parametric sigmoidal model, which was applied by Tichopad et al. [30] to study the kinetic parameters of a polymerase chain reaction, is extended with a fifth parameter to fit the online monitored temporal development of the scattered light intensity signal:

1 $$ {SL}_{fit}( t)= a+\frac{b}{1+ \exp \left(-\frac{t- c}{d}\right)}+ e\cdot t $$

**Fig. 3:**
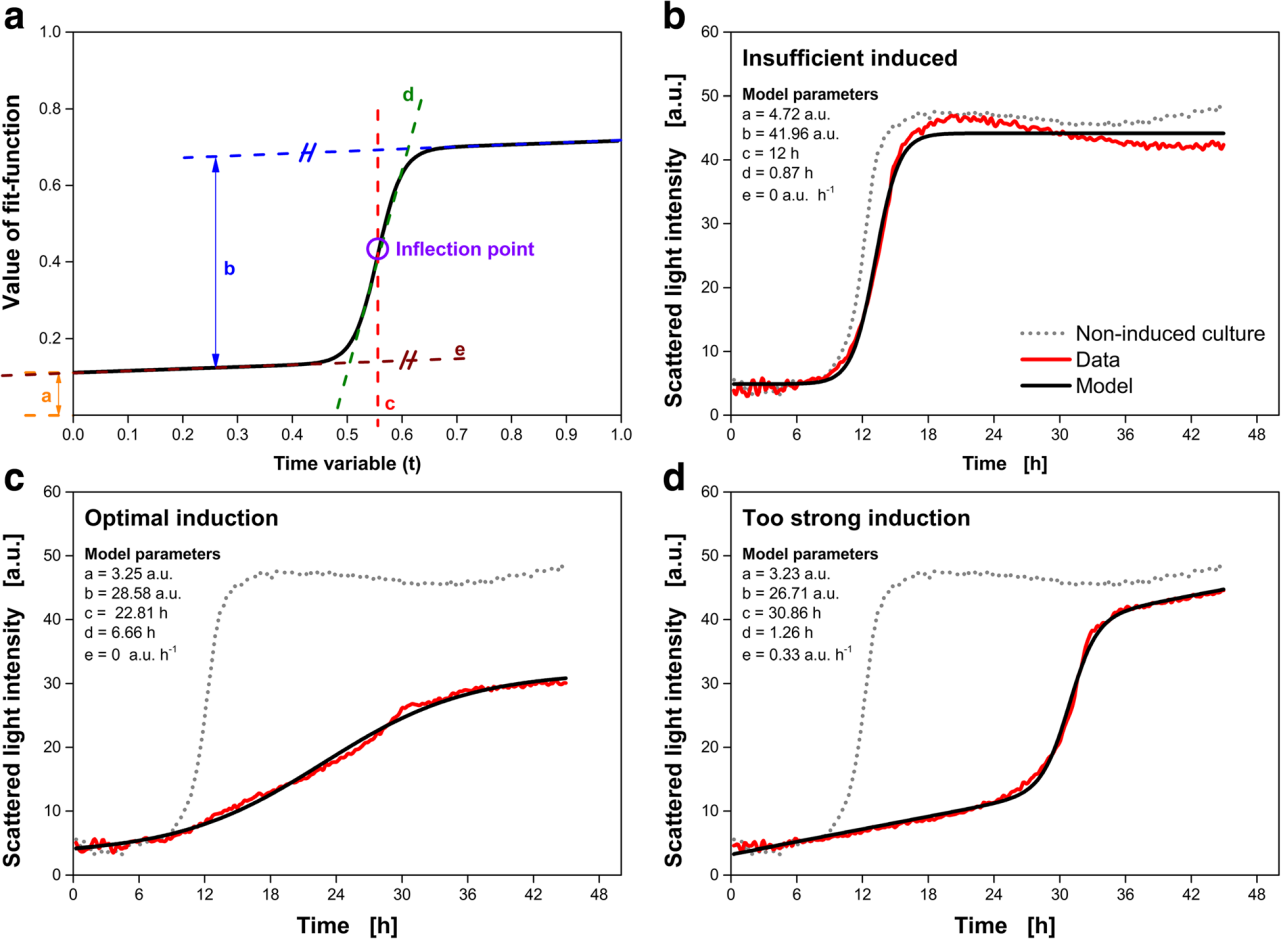
Analysis of the scattered light intensity patterns following a semi-mechanistic approach based on an extended sigmoidal function (**a**). The extended sigmoidal function consists of five parameters (*a*-*e*) and is described by Eq. 1. In this model, *a* [a.u.] represents the lowest scattered light intensity, *b* [a.u.] corresponds to the hub of the curve, *c* [h] is the time of the inflection point (value on x-axis), *d* [h] is a parameter inversely proportional to the slope of the sigmoidal curve, and *e* [a.u. h^−1^] is the linear slope. Exemplarily, measured scattered light intensity signals and model data over time are shown belonging to different classification according to Fig. 2: insufficient induction (**b**), optimal induction (**c**) and too strong induction (**d**). The corresponding model parameters are given in the figure. As reference, the pattern of a non-induced culture is presented

In Eq. 1, *SL*_*fit*_(*t*) [a.u.] is the value of the function computed at the time *t* [h]. *a* [a.u.] is the lowest scattered light intensity, *b* [a.u.] is the difference between minimum and maximum scattered light intensity, *c* [h] is the time of the inflection point of the curve and *d* [h] represents a parameter inversely proportional to the slope of the sigmoidal fraction. The meaning of each parameter is visualized in Fig. 3a. This function can easily be transformed by its parameters in two extremes: a step-function or a continuous linear increasing function.

In the past, sigmoidal functions were already often used to describe biomass formation during culture [31]. The parameters of a sigmoidal function are usually mathematical parameters and do not provide any mechanistic parameter of biological interest. Zwietering et al. [31] modified several model equations (Logistic, Gompertz, Richards, Schnute, and Stannard) so that each parameter contains biological relevant information (maximum growth rate and duration of the lag-phase). However, this modification led to more complicated mathematical expressions. Such a kind of modification was not carried out in the present work, because the target of this study is not the determination of kinetic parameters. Instead, this work aims at an easy to use mathematical method to identify insufficient, optimal and too strong induction. In addition, an empirical model to predict the relative expression performance should be derived just based on the scattered light intensity trajectory. Therefore, a modification (and complication) of the applied fitting function is not necessary.

The equation of the extended sigmoidal function (Eq. 1) includes five parameters (*a*- *e*). In Fig. 3a, the function over time is shown and the meaning of each parameter is illustrated. Additionally, the inflection point of the sigmoidal curve is highlighted. In Fig. 3b-d, the scattered light signal of exemplarily cultures belonging to insufficient induction (Fig. 3b), optimal induction (Fig. 3c) and too strong induction (Fig. 3d) are presented (red lines). As reference in each figure, gray dashed lines present the temporal development of the scattered light of a non-induced culture. The corresponding fits of *SL*_*fit*_(*t*) are shown as black lines. In the upper left corner of each figure, the fitting parameters found are given. It is clearly visible that the fits represents the online monitored temporal development of the scattered light very good.

**Fig. 4:**
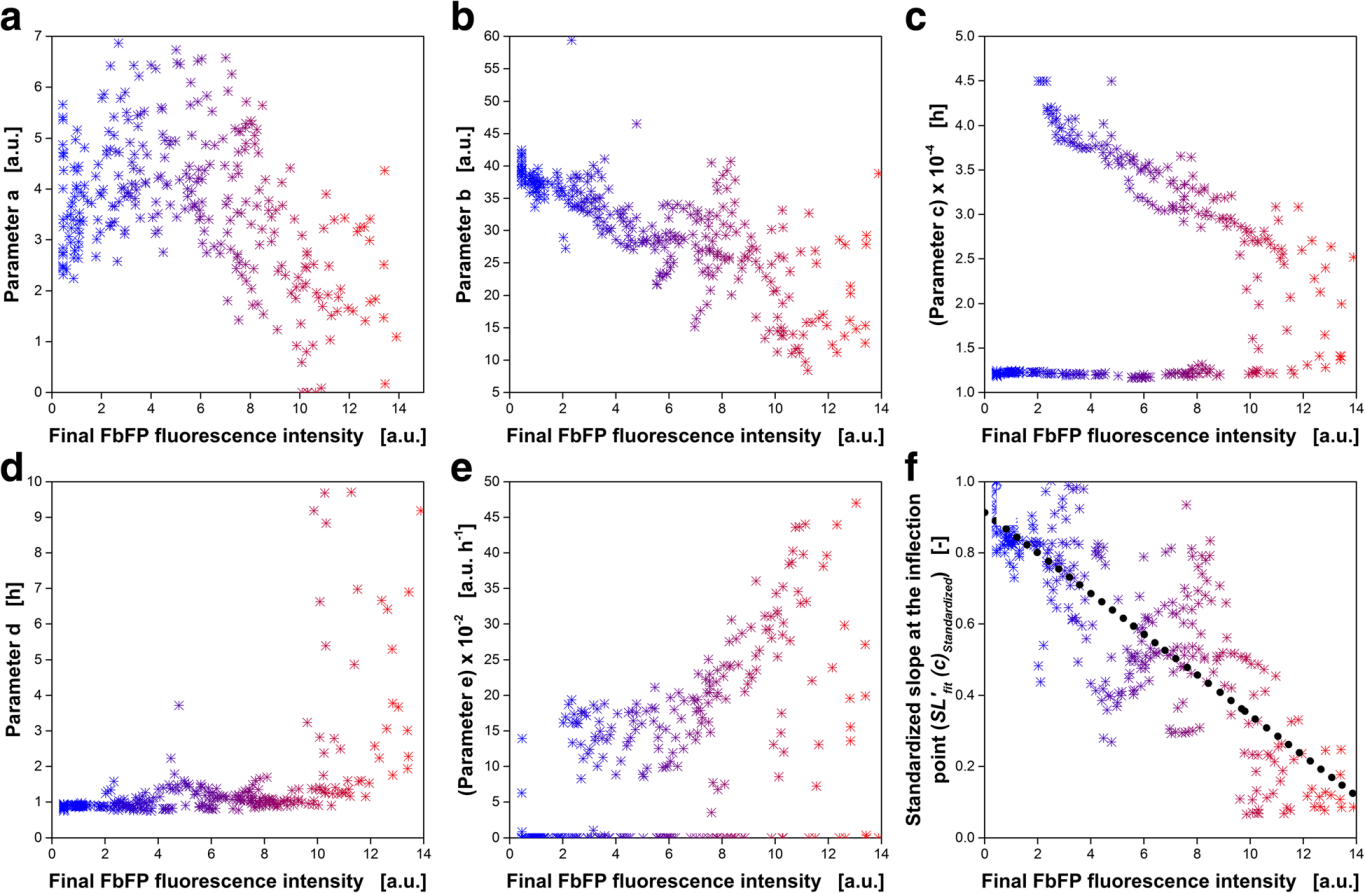
Investigation of correlations between the final FbFP fluorescence intensity and parameters of the extended sigmoidal function (Eq. 1). In each plot, every symbol represents one complete *E. coli* Tuner(DE3)/pRhotHi-2-LacI-EcFbFP culture (dataset A). Four single cultures of the initial dataset of 303 cultures introduced in Fig. 1 were removed due to bad fit to the extended sigmoidal function. For all remaining 299 fits, regression coefficients (R^2^) higher than 0.96 are reached. The color represents the final FbFP fluorescence intensity and corresponds to the color-coding in Fig. 1. **a**-**e** Values of the five parameters (*a*-*e*) of the extended sigmoidal function for each culture (Eq. 1). **f** Calculated slope at time of the inflection point (parameter *c*) for each culture (Eq. 3). The slope is standardized according to Eq. 7. The black dotted line represents a linear regression (R^2^ = 0.66). Cultivation conditions: 48 round deep-well MTP without optodes, V_L_ = 800 μL, *n* = 1000 rpm, shaking diameter d_0_ = 3 mm, 30 °C

Correlations between two variables are easily detectable by means of scatter plots. In the present work, scatter plots are used to identify whether any parameter of *SL*_*fit*_(*t*) correlates with the final FbFP fluorescence intensity. Therefore, *SL*_*fit*_(*t*) was fitted to the temporal development of the scattered light of each of the 303 cultures. For the following investigations, only data of fits with regression coefficients (R^2^) greater than 0.96 are used. This restriction results in exclusion of 4 cultures and, thus, 299 cultures remain in the dataset. In Fig. 4, the obtained fitting parameters are plotted over the final FbFP fluorescence intensity. Fig. 4a-e correspond to the fitting parameters *a*- *e*. Again, the color-coding introduced in Fig. 1 was applied.

Parameter *a* (Fig. 4a) decreases slightly with increasing final FbFP fluorescence intensity. However, the scatter is very large compared with the overall decrease. In addition, parameter *a* represents the minimum scattered light intensity (compare Fig. 3a) and from a theoretical point of view, a correlation does not make sense. Without any measurement errors and (non-influenceable) external disturbances, the minimum scattered light intensity should be the same for all cultures.

Parameter *b* (Fig. 4b) indicates the hub of the scattered light intensity during cultivation. Since the hub in scattered light intensity over time represents the overall biomass formation, a correlation is very likely and found in Fig. 4b. The final FbFP fluorescence intensity is increasing with decreasing values of parameter *b*. In cultures with smaller hub (small values of parameter *b*), the overall biomass formation is reduced. This means that the consumed carbon source is increasingly used for product expression instead of biomass formation. However, at low values of parameter *b*, the correlation becomes noticeably worse.

Parameter *c* (Fig. 4c), *d* (Fig. 4d) and *e* (Fig. 4d) are obviously non-correlated with the final FbFP fluorescence intensity. Especially, for parameter *d* this finding is surprising because parameter *d* is inversely proportional to the slope of the sigmoidal fraction. With decreasing values of parameter *d*, the slope is increasing and, thus, the curve rises more quickly. Increasing values of parameter *d* result in a more linear rise of the sigmoidal fraction of *SL*_*fit*_(*t*). The effect of parameter *d* on the slope of the sigmoidal fraction also can nicely be seen in Fig. 3b-d by the respective model parameters in the upper left of each figure.

To determine the slope of the extended sigmoidal function at any time (*t*) the first derivative of Eq. 1 is calculated:

2 $$ {SL}_{fit}^{\prime }( t)=\frac{b}{2\cdot d\cdot \cosh \left(-\frac{t- c}{d}\right)+2\cdot d}+ e $$

${SL}_{fit}^{\prime }(t)$ [a.u. h^−1^] is the derivative of the extended sigmoidal function (*SL*_*fit*_(*t*); Eq. 1) at the time *t* [h] and, thus, represents the slope of the function at any time (*t*).

The slope at the center of the sigmoidal fraction is probably the best representative to identify whether the sigmoidal fraction is rising moderate (linear) or fast (exponential). A small slope at that point indicates moderate (linear) increase, while a great slope represent an exponential rise. The center of the sigmoidal fraction is equal to the time of the inflection point (parameter *c*). Thus, the slope at the time *c* needs to be determined (${SL}_{fit}^{\prime }(c)$), which is expected to correlate with the final FbFP fluorescence intensity. At the inflection point (*t* = *c*), Eq. 2 simplifies with cosh(0) = 1 to:

3 $$ {SL}_{fit}^{\prime }( c)=\frac{b}{4\cdot d}+ e $$

${SL}_{fit}^{\prime }(c)$depends on the online measurement device because the slope is given in arbitrary units per hour (a.u. h^−1^). To overcome the dependency on the online measurement device, ${SL}_{fit}^{\prime }(c)$ is standardized to a (non-induced) reference culture and a dimensionless characteristic parameter is received (${SL}_{fit}^{\prime }{(c)}_{Standardized}$; Eq. 7). In Fig. 4f, ${SL}_{fit}^{\prime }{(c)}_{Standardized}$ is presented over the final FbFP fluorescence intensity. As expected, with decreasing ${SL}_{fit}^{\prime }{(c)}_{Standardized}$ the measured final FbFP fluorescence intensity is increasing. The black dashed line represents a linear correlation.

Clustering, as it was conducted via PCA (Fig. 2c), requires two characteristic parameters. Thus, a second characteristic parameter needs to be found to distinguish between the different groups. At that point, it is worth to reflect which information is contained in each of the two PCs. The score values of the second PC contain information about the expression performance (Fig. 2c). Exactly this information is also covered by ${SL}_{fit}^{\prime }{(c)}_{Standardized}$ (Fig. 4f). For discrimination between the different group of induction, whether the culture belongs to insufficient, optimal or too strong, turned out to be contained mostly in the score values of the first PC. Consequently, a second characteristic parameter based on *SL*_*fit*_(*t*) should also represent this information. The most obvious differences between the three groups is the time when the inflection point appears (compare Fig. 3). This information is directly given by parameter *c* of *SL*_*fit*_(*t*). To achieve a more general validity for comparison between cultures conducted at different culture conditions, parameter *c* is standardized to (non-induced) reference culture according to Eq. 6. Like ${SL}_{fit}^{\prime }{(c)}_{Standardized}$, *c*_*Standardized*_ is a dimensionless characteristic parameter.

With ${SL}_{fit}^{\prime }{(c)}_{Standardized}$ and *c*_*Standardized*_ two characteristic parameters are found which can be used for clustering. These two parameters offer the advantage to be easily computable after fitting the monitored temporal development of the scattered light intensity by *SL*_*fit*_(*t*). Advanced statistical or mathematical knowledge is not required. Furthermore, a dependency on the measurement system and the culture conditions is neglected by standardizing the values to (non-induced) reference cultures. To confirm the general validity, six additional datasets were investigated (in total 322 additional *E. coli* cultures). The overall dataset (621 cultures) consists of data received from two BioLector devices, at four temperatures (28 °C, 30 °C, 34 °C, and 37 °C), three *E. coli* host strains (Tuner(DE3), BL21(DE3), and BL21-Gold (DE3)) and two investigated products (FbFP and Cellulase (celA2)). For each culture, *SL*_*fit*_(*t*) was fitted to the temporal development of the scattered light. Subsequently, ${SL}_{fit}^{\prime }{(c)}_{Standardized}$ as well as *c*_*Standardized*_ were calculated.

**Fig. 5:**
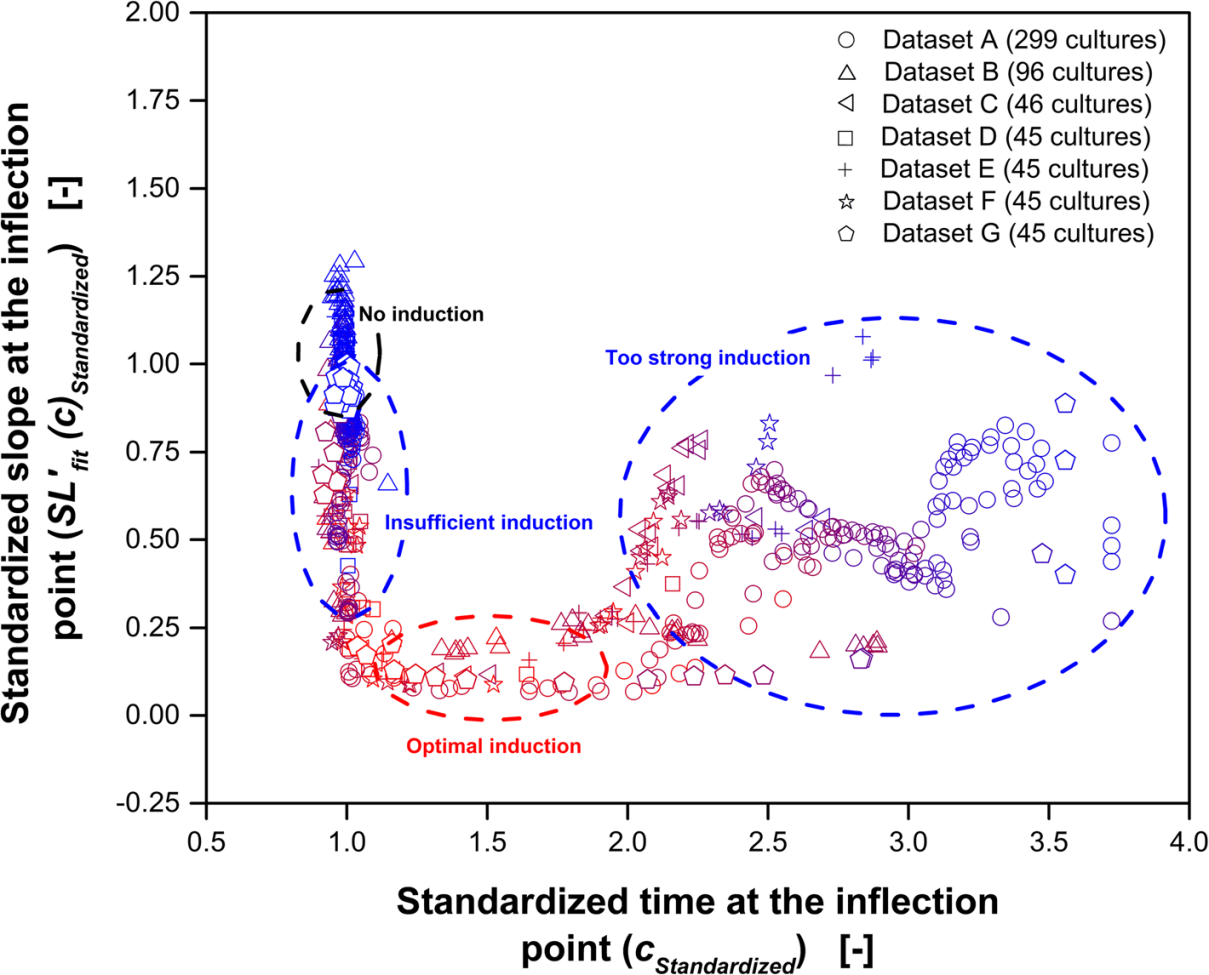
Classification of expression performance based on parameters obtained from fitting the extended sigmoidal function (Eq. 1) to scattered light intensity datasets. Standardized slope at the inflection point (${SL}_{fit}^{\prime }{(c)}_{Standardized}$; Eq. 7) is plotted over the standardized time at the inflection point (*c*_*Standardized*_; Eq. 6). Cultures were conducted under quite different conditions in two BioLector devices, two types of microtiter plates (48-well and 96-well) and at four temperatures (28 °C, 30 °C, 34 °C and 37 °C) with three different *E. coli* host strains (Tuner(DE3), BL21(DE3) and BL21-Gold(DE3)). The detailed culture conditions are given in Table 1. In total 621 different cultures are presented and each symbol represents one complete culture. Within each dataset, red represents the best expression performance while blue indicates bad expression performance. The cultures were classified in accordance to Fig. 2c in four clusters: no induction, insufficient induction, optimal induction and too strong induction. The specific ranges are indicated by ellipses

In Fig. 5, ${SL}_{fit}^{\prime }{(c)}_{Standardized}$ is presented over *c*_*Standardized*_ for all 621 cultures. Each point represents one whole culture. A color-coding, similar to the color-coding used in the previous figures is used. Pure red indicates the best expression performance and blue represents a weak expression performance. Because enzyme activity and FbFP fluorescence intensity cannot directly be compared with each other and, the fluorescence itself depends on the temperature [32], each dataset was decoded separately. The datasets are distinguishable by different symbols. All cultures follow an obvious general trend. The best relative expression performance is obtained in cultures showing the following properties:

1. $$ {SL}_{fit}^{\prime }{(c)}_{Standardized}<0.25 $$
2. 1.1 < *c*_*Standardized*_ < 1.9

The area of non-induced cultures as well as cultures with insufficient, optimal, and too strong induction are highlighted and labeled in Fig. 5. Discrimination between non-induced cultures and cultures with insufficient induction is not readily possible based on this method. Optimal induction is defined for cultures meeting the two criteria above. Cultures showing values of ${SL}_{fit}^{\prime }{(c)}_{Standardized}$ greater than 0.25 and *c*_*Standardized*_ smaller than 1.1 tend to be insufficient induced. For those cultures, an increased amount of inducer concentration and/or an earlier induction are expected to result in better expression performance. Too strong induction is found for cultures with *c*_*Standardized*_ greater than 2. In this case, it is advisable to reduce the induction level and/or to use at later point in time for induction. Although no direct proposal for absolute changes of the induction parameters (time-point of induction or IPTG concentration) can be given based on this method, valuable information for process optimization are obtained. Especially in strain screening experiments, non-optimal induction parameters can easily be identified. Optimized induction parameters might result in significantly increased expression performance of the corresponding clone and too early exclusion of this clone could be avoided.

### Prediction of standardized expression performance based on ${SL}_{fit}^{\prime }{(c)}_{Standardized}$ and *c*_*Standardized*_

In the previous section, clustering of *E. coli* cultures into insufficient, optimal and too strong induction was performed based on the values of ${SL}_{fit}^{\prime }{(c)}_{Standardized}$ and *c*_*Standardized*_. The values are obtained just by fitting an extended sigmoidal function to the temporal development of the scattered light. A model that is able to predict the expression performance of an *E. coli* culture would be very valuable. Such a model would particularly be helpful if the determination of the product concentration requires elaborated laboratory examination. In addition, it would become possible to assess whether, and if so, in which order of magnitude the expression performance can be improved by optimizing the induction parameters without additional experiments.

In Fig. 4f, the linear correlation (black dashed line) already represents a very simple model to predict the expression of FbFP of *E. coli* Tuner(DE3) at 37 °C. As indicated by the low regression coefficient (R^2^) of 0.66 (Fig. 4f), based on ${SL}_{fit}^{\prime }{(c)}_{Standardized}$ just a rough prediction of the final FbFP fluorescence intensity is possible. To improve the quality of the prediction model, in addition the second characteristic parameter (*c*_*Standardized*_) will be used.

For the development of the empirical prediction model, all datasets (A-G) are used. As indicated in Table 1, a wide range of culture condition is covered by these datasets. However, the fluorescence intensity is temperature depended [32] and different culture temperatures are investigated (compare Table 1). The expressed product is either directly measurable via fluorescence (FbFP) or the enzyme activity (Cellulase celA2) has to be detected via offline assay. To allow for comparison of all datasets, the introduction of a standardized expression performance (*EP*_*Standardized*_) is necessary. Weak (or almost none) expression performance will be indicated by *EP*_*Standardized*_-values close to 0. The best-measured expression performance should be represented by a value of 1 by *EP*_*Standardized*_. As basis for the empirical model, an equation including main effects, linear and quadratic interactions is applied. The coefficients were determined using the method of least squares. The following equation was found to predict *EP*_*Standardized*_ based on *c*_*Standardized*_ and ${SL_{fit}^{\prime }(c)}_{Standardized}$:

4 $$ {EP}_{\mathrm{Standardized}}=0.76+0.37\cdot {c}_{\mathrm{Standardized}}-0.14\cdot {c}_{\mathrm{Standardized}}^2-1.14\cdot {S{ L}_{fit}^{\prime }(c)}_{\mathrm{Standardized}}+0.27\cdot {c}_{\mathrm{Standardized}}\cdot {S{ L}_{fit}^{\prime }(c)}_{\mathrm{Standardized}} $$

**Table 1:** Summary of the investigated datasets

Dataset	Cultures	MTP	*E. coli* host train	Product	Temp.	ID-No of Device	Source
A	303	48-well	Tuner(DE3)	FbFP	30 °C	1	Wandrey et al. [33]
B	96	96-well	BL21(DE3)	FbFP	37 °C	2	Huber et al. [10]
C	46	48-well	Tuner(DE3)	FbFP	28 °C	2	
D	45	48-well	Tuner(DE3)	FbFP	30 °C	2	
E	45	48-well	Tuner(DE3)	FbFP	34 °C	2	
F	45	48-well	Tuner(DE3)	FbFP	37 °C	2	
G	45	48-well	BL21-Gold (DE3)	Cellulase (celA2)	37 °C	2	

Because the quadratic interaction of the standardized slope at the inflection point (${{SL_{fit}^{\prime }(c)}_{Standardized}}^2$) did not improve the model quality, this factor is not included in the model (Eq. 4). The regression coefficient (R^2^) was determined with 0.74 and, thus, is significantly better than the linear regression (R^2^ = 0.66), presented in Fig. 4f which is just based on ${SL_{fit}^{\prime }(c)}_{Standardized}$.

**Fig. 6:**
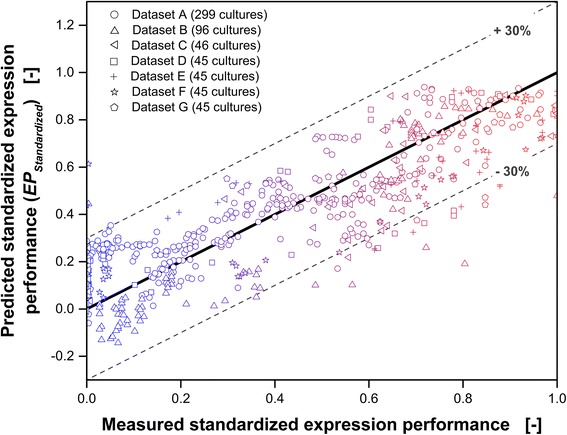
Comparison of predicted (Eq. 4) and measured standardized expression performance based on the standardized slope and time at the inflection point as defined in Fig. 3a. The expression performance is calculated according to the empirical correlation (Eq. 4). The highest expression performance is represented by a value of 1 and the lowest expression performance is indicated by 0. The dashed lines indicate a standard deviation of ±30%. The detailed culture conditions are given in Table 1. In total 621 different cultures are presented and each symbol represents one complete culture. Red represents the best expression performance while blue indicates bad expression performance

In Fig. 6, *EP*_*Standardized*_ predicted according to Eq. 4 is plotted over the measured *EP*_*Standardized*_. All 621 cultures are shown. The datasets are indicated by different symbols. Again, the color-coding introduced in Fig. 5 is used. Red represents the best expression performance and becomes more bluish with decreasing expression performance. The color-coding corresponds to values of *EP*_*Standardized*_ between 0 (pure blue) and 1 (pure red). More than 95% of the measured standardized expression performances fit to the calculated values with an accuracy of 30% (gray dashed lines indicate a standard deviation of ±30%).

It is clearly visible that it is possible to predict the standardized expression performance of *E. coli* cultures just based on the temporal development of the scattered light (Eq. 4). This method is particularly useful if the expressed product cannot directly be measured via fluorescence. Complex HPLC analysis, ELISA or enzyme assays can be avoided for a first screen and the experimental evaluation becomes much easier and faster. Because the empirical prediction model is based on standardized dimensionless characteristic parameters of the temporal development of the scattered light, the model can be applied to different BioLector devices, different *E. coli* strains and expressed products and with various culture conditions.

## Conclusions

In a first approach, correlation between the temporal development of the scattered light during *E. coli* cultures and the amount of expressed fluorescent protein (FbFP) was identified via visualization. It turned out that good expression performances are indicated by linear increasing scattered light intensities after induction. Due to principal component analysis (PCA) it was found that already the score values of the first two principal components (PCs) are sufficient, to represent an entire culture. The scores of the first PC allow for discrimination between insufficient, optimal and too strong induction. The scores of the second PC mostly give information about the expression performance.

The PCs were calculated based on scattered light measurements with equal discrete time steps and, thus, the PCA-model depends to a certain extent on the measurement setup and the chosen culture conditions. To overcome this restriction, a more mechanistically approach was followed. Therefore, an extended sigmoidal function (*SL*_*fit*_(*t*)) is fitted to the temporal development of the scattered light of each culture. Based on the result of the fits, two characteristic parameters are found: *c*_*Standardized*_ and ${SL}_{fit}^{\prime }{(c)}_{Standardized}$. *c*_*Standardized*_ is the standardized time of the inflection point of the sigmoidal fraction. ${SL}_{fit}^{\prime }{(c)}_{Standardized}$ is the standardized slope of the scattered light intensity signal at the inflection point. As reference for standardization, the temporal development of the scattered light of a non-induced culture is used. These characteristic parameters can be used for clustering the cultures by their quality of induction as well as for prediction of a standardized expression performance (*EP*_*Standardized*_). More than 95% of *EP*_*Standardized*_ were in agreement with the measured values with an accuracy of 30%. Considering the fact that data from very different cultures were evaluated, this accuracy can be considered as satisfactory. Due to the standardization to a (non-induced) reference culture, a more general validity is achieved and the method was successfully applied to numerous *E. coli* cultures with various culture conditions. In total, the temporal development of the scattered light of 625 *E. coli* cultures (three host strains: Tuner(DE3), BL21(DE3), and BL21-Gold (DE3)) expressing either FbFP or cellulase (celA2) was investigated. The combined datasets include cultures conducted in two different online measurement devices with two types of MTPs (48- and 96 wells), at four temperatures (28 °C, 30 °C, 34 °C, and 37 °C). To the best of our knowledge, this is the first work presenting a method for the general prediction of expression performance of *E. coli* based solely on the temporal development of scattered light signals. The presented methodology is well suited to enhance induction optimization and speed-up bioprocess development. In future, it has to be investigated whether this methodology can be extended also to auto-induction media and even to other gram-negative or gram-positive expression host strains.

## Methods

### Dataset used for investigation

In this work, seven datasets (A-G) were investigated. Partially, datasets have already been published before. The cultures contained in dataset A were published by Wandrey et al. [33]. Dataset B was obtained from Huber et al. [10]. Dataset C-G were not published before. For online monitoring of the cultures contained in dataset C-G, the same online measurement device as presented by Huber et al. [10] was used. The number of investigated cultures as well as the culture conditions of each dataset are summarized in Table 1.

### Microorganism and media

For the investigations in dataset A and C-F, *Escherichia coli* Tuner(DE3)/pRhotHi-2-LacI-EcFbFP was cultivated in 48-well FlowerPlates (MTP-48-B, lot 1404 & 1509, m2p-labs, Baesweiler, Germany). Two pre-cultures (first: complex TB medium; second: synthetic Wilms-MOPS medium) were conducted in 250 mL shake flask (37 °C; shaking frequency (*n*): 350 rpm; shaking diameter (*d*_0_): 50 mm; filling volume (*V*_*L*_): 10 mL). Synthetic modified Wilms-MOPS medium with 20 g L^−1^ glucose was used for main culture. Further details about the media composition is given elsewhere [33, 34].

In dataset G, cultures of *Escherichia coli* BL21-Gold (DE3) pET-t7-CelA2 were investigated. Again, two pre-cultures (first complex, second synthetic medium) and synthetic modified Wilms-MOPS medium with 20 g L^−1^ glucose for main culture were used. The time of induction was chosen between 1 and 10 h with IPTG concentrations between 50 and 1000 μM.

In dataset B *Escherichia coli* BL21(DE3) pRhotHi-2-EcFbFP was investigated. Synthetic MDG mineral medium [35] with 5 g L^−1^ glucose as carbon source was used for the main cultures in a 96-well MTP (μClear, Greiner Bio-One, Frickenhausen, Germany). Further information are given elsewhere [10].

### Measurement setup

All cultures were online monitored by measurement devices according the BioLector concept [15, 16]. This technology allows for fluorescence and scattered light measurements through the transparent bottom in each well of continuously orbitally shaken MTPs. Because the shaking movement is not interrupted during measurement, the risk of (temporary) oxygen limitations and cell sedimentation is significantly reduced. As indicated in Table 1, two different measurement devices with varying optics were utilized. The setup of “device 1” was developed in-house and is described in more detail by Wandrey et al. [33]. Up to four parallel MTPs can be monitored in parallel with this device. Details of “device 2” are given by Huber et al. [10]. In this custom made setup, the BioLector is integrated into the worktable of a liquid handling system allowing process automation. For a comparison of the scattered light level measured with this device and optical density, a proportionality factor of 4 must be considered. For example, a scattered light level of 20 a.u. corresponds roughly to an optical density (OD_600nm_) of 5. Due to the different optical measurement systems, variations of the measured absolute values (arbitrary units) in scattered light and fluorescence are unavoidable. However, to enable comparison of data received from both online measurement devices, mathematical methods to standardize the results are applied as described in the following sections.

### 4-MUC assay

The final endoglucanase activity of Cellulase (celA2) in dataset G was offline measured by means of the fluorescence-based 4-methylumbelliferyl-β-D-cellobioside (4-MUC) assay [36]. Small changes, as reported by Mühlmann et al. [17] were applied. The 4-MUC assay was conducted after reaching the stationary phase (indicated by the scattered light signal) and, thus, the time-point of analysis varied due to different culture conditions between 10 and 24 h.

### Software

All calculations were performed in MATLAB (Version R2016a 9.0.0.341360, The MathWorks, Inc., Natik, USA). Figures were created with OriginPRO 2016G (Version b9.3.226, OriginLab Corperation, Northampton, USA).

### Standard normal variate transformation

To enhance the quality of data used for principal component analysis (PCA), data pre-processing is often conducted. In the literature, different methods for data pre-processing are presented and discussed [29, 37, 38]. In this contribution, the standard normal variate (SNV) transformation is applied to the scattered light intensity signals that were acquired during cultivation [39]. The scattered light intensity signal of each culture is processed individually. From each measured scattered light value (*SL*(*t*)) the scattered light intensity signal mean of the entire cultivation ($\overset{-}{SL}$) is subtracted and divided by the standard deviation of the scattered light intensity signal of the entire cultivation (*std*(*SL*)):

5 $$ SNV\left( SL(t)\right)=\frac{SL(t)-\overset{-}{SL}}{std(SL)} $$

### Standardization of characteristic model parameters by referencing to a non-induced culture

As already mentioned above, data obtained from two online measurement devices were investigated in this work. Even if the identical sample is investigated in both devices different absolute values (arbitrary units) are measured due to different optics. Furthermore, the type of applied MTPs (48-well and 96-well plates) influence the course of the light beam and, thus, influence the measurement. From dataset to dataset, also different culture conditions are investigated. These changes have significant impact on the cultivation. For example, a temperature decrease extends the lag phase of the culture. Therefore, the measured data needs to be standardized to realize a comparison between all investigated datasets. A non-induced culture is used as reference. The dimensionless standardized time of the inflection point (*c*_*Standardized*_) is calculated according to Eq. 6:

6 $$ {c}_{Standardized}=\frac{c}{c_{non- induced}} $$

${c}_{non- induced}$ is the time of the inflection point (parameter *c* of the extended sigmoidal function; see Fig. 3a) of a non-induced culture (reference).

Following the same approach, the dimensionless standardized slope at the time of the inflection point (${SL}_{fit}^{\prime }{(c)}_{Standardized}$) is calculated according to Eq. 7:

7 $$ {SL}_{fit}^{\prime }{(c)}_{Standardized}=\frac{{SL_{fit}}^{\prime }(c)}{{SL_{fit}\prime (c)}_{non- induced}} $$

*SL*_*fit*_^′^(*c*) [a.u. h^−1^] is the slope at the inflection point of the curve of the currently investigated culture. ${SL}_{fit}^{\prime }{(c)}_{non- induced}$ [a.u. h^−1^] is the slope at the inflection point of the curve of a non-induced culture (reference culture).

## Additional file

Additional file 1: Figure S1. Monitored scattered light (A) and FbFP fluorescence intensity signals (B) during 303 *E. coli* Tuner(DE3)/pRhotHi-2-LacI-EcFbFP cultures with varying times of induction (0.5–16 h) and concentrations of IPTG (0–1000 μM). (A) The time span of induction is highlighted by the gray area. (B) The final FbFP fluorescence intensity defines the color-coding. The weakest expression (≙ minimum final FbFP fluorescence intensity) is presented in blue and becomes more reddish with increasing final fluorescence intensity. The maximum final fluorescence intensity is presented in pure red. The presented cultures were conducted in a total of eight MTPs. The investigated cultures are referred to as dataset A in Table 1. Cultivation conditions: 48 round deep-well MTP without optodes, VL = 800 μL, *n* = 1000 rpm, shaking diameter d0 = 3 mm, 30 °C. (PDF 437 kb)

## Abbreviations

4-MUC: 4-methylumbelliferyl-β-D-cellobioside
*a*: Lowest scattered light intensity of extended sigmoidal fit function [a.u.]
*b*: Difference between lowest and maximum scattered light intensity of extended sigmoidal fit function [a.u.]
*c*: Time of inflection point of extended sigmoidal fit function [h]
*c*_*non* − *induced*_: Time of inflection point of extended sigmoidal fit function of a (non-induced) reference culture [h]
*c*_*Standardized*_: Standardized time of inflection point of extended sigmoidal fit function [−]
*d*: Parameter inversely proportional to the slope of the sigmoidal fraction of the extended sigmoidal function [h]
*d*_0_: Shaking diameter [mm]
*e*: Slope of the linear fraction before and after the extended sigmoidal function [a.u. h^‑1^]
FbFP: Flavin-mononucleotide-based fluorescent protein
GFP: Green fluorescent protein
IPTG: Isopropyl β-D-1-thiogalactopyranoside
MTP: Microtiter plate
*n*: Shaking frequency [rpm]
PC: Principal component
PCA: Principal component analysis
*t*: Time [h]
*SL*(*t*): Temporal development of the scattered light during culture [a.u.]
$\overset{-}{SL}$: Scattered light intensity signal mean of an entire cultivation [a.u.]
*SL*_*fit*_(*t*): Extended sigmoidal function to fit scattered light intensity signal [a.u.]
${SL}_{fit}^{\prime }(t)$: Derivative of the extended sigmoidal function (*SL*_*fit*_(*t*)) [a.u. h^‑1^]
${SL}_{fit}^{\prime }(c)$: Slope of the extended sigmoidal function at the inflection point [a.u. h^‑1^]
*SL*_*fit*_ ′ (*c*)_*non* − *induced*_: Slope of the extended sigmoidal function at the inflection point of a (non-induced) reference [a.u. h^‑1^]
${SL}_{fit}^{\prime }{(c)}_{Standardized}$: Standardized slope of the extended sigmoidal function at the inflection point [−]
*SNV*(*SL*(*t*)): Standard normal variate (SNV) transformation of the scattered light intensity signal [−]
*std*(*SL*): Standard deviation of the scattered light intensity signal of an entire cultivation [a.u.]
*V*_*L*_: Filling volume [mL]
