# Supplementary material for: Prediction of Escherichia coli expression performance in microtiter plates by analyzing only the temporal development of scattered light during culture
Source: J Biol Eng. 2017 Jul 3;11:20. doi: 10.1186/s13036-017-0064-5 (PMC5494857; doi:10.1186/s13036-017-0064-5)
Supplement: Additional file 1: Figure S1. — Monitored scattered light (A) and FbFP fluorescence intensity signals (B) during 303 E. coli Tuner(DE3)/pRhotHi-2-LacI-EcFbFP cultures with varying times of induction (0.5–16 h) and concentrations of IPTG (0–1000 μM). (A) The time span of induction is highlighted by the gray area. (B) The final FbFP fluorescence intensity defines the color-coding. The weakest expression (≙ minimum final FbFP fluorescence intensity) is presented in blue and becomes more reddish with increasing final fluorescence intensity. The maximum final fluorescence intensity is presented in pure red. The presented cultures were conducted in a total of eight MTPs. The investigated cultures are referred to as dataset A in Table 1. Cultivation conditions: 48 round deep-well MTP without optodes, VL = 800 μL, n = 1000 rpm, shaking diameter d0 = 3 mm, 30 °C. (PDF 437 kb) [file 13036_2017_64_MOESM1_ESM.pdf]

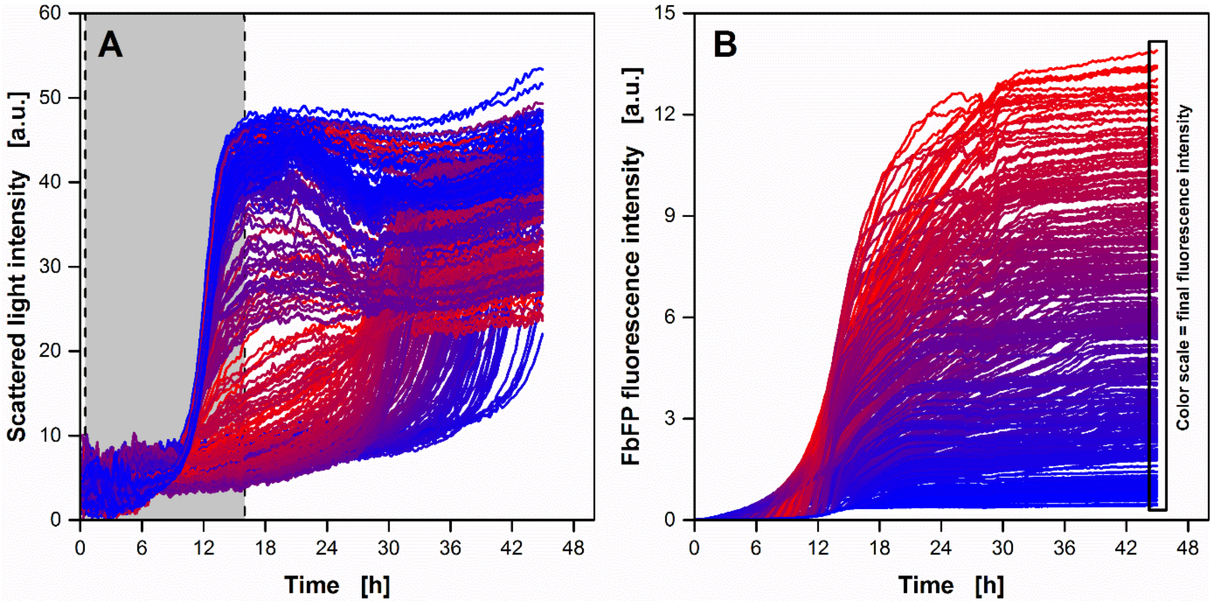

Additional Figure 1: Monitored scattered light (A) and FbFP fluorescence intensity signals (B) during 303 *E. coli* Tuner(DE3)/pRhotHi-2-LacI-EcFbFP cultures with varying times of induction (0.5–16 h) and concentrations of IPTG (0–1000 µM). (A) The time span of induction is highlighted by the gray area. (B) The final FbFP fluorescence intensity defines the color-coding. The weakest expression (≙ minimum final FbFP fluorescence intensity) is presented in blue and becomes more reddish with increasing final fluorescence intensity. The maximum final fluorescence intensity is presented in pure red. The presented cultures were conducted in a total of eight MTPs. The investigated cultures are referred to as dataset A in Table 1. Cultivation conditions: 48 round deep-well MTP without optodes, V_L = 800 µL, n = 1000 rpm, shaking diameter d_0 = 3 mm, 30 °C
